# Usefulness of ^18^F-Fluorodeoxyglucose Positron Emission Tomography for Follow-Up of 13-*cis*-Retinoic Acid Treatment for Residual Neuroblastoma After Myeloablative Chemotherapy

**DOI:** 10.1097/MD.0000000000001290

**Published:** 2015-08-07

**Authors:** Yuya Sato, Hidemitsu Kurosawa, Setsu Sakamoto, Shigeko Kuwashima, Teisuke Hashimoto, Kentaro Okamoto, Takashi Tsuchioka, Keitaro Fukushima, Osamu Arisaka

**Affiliations:** From the Department of Pediatrics (YS, HK, KF, OA), Dokkyo Medical University; Positron Emission Tomography Center (SS), Dokkyo Medical University Hospital; Department of Radiology (SK, TH), Dokkyo Medical University; and First Department of Surgery (KO, TT), Dokkyo Medical University, Japan.

## Abstract

13-*cis*-retinoic acid (13-*cis*-RA) treatment is used as a second-line treatment for residual or recurrent neuroblastoma. However, determining the duration of 13-*cis*-RA treatment for residual and recurrent neuroblastoma can be a problem because it is difficult to evaluate the effectiveness of the treatment.

We performed 13-*cis*-RA treatment to remove residual active neuroblastoma cells in an 8-year-old boy with stage 4 neuroblastoma that developed from a left sympathetic ganglion and had been treated with chemotherapy, surgery, autologous peripheral blood stem-cell transplantation, and radiotherapy. ^18^F-fluorodeoxyglucose positron emission tomography (^18^F-FDG-PET) and iodine-123 metaiodobenzylguanidine (^123^I-MIBG) scintigraphy obtained immediately before 13-*cis*-RA treatment both showed positive findings in the area of the primary lesion. At 18 months after 13-*cis*-RA treatment, there was accumulation on ^123^I-MIBG scintigraphy but no uptake on ^18^F-FDG-PET, and 13-*cis*-RA treatment was suspended. The patient has been in complete remission for 3 years. In comparing the effectiveness of the 2 imaging modalities for monitoring the response to 13-*cis*-RA treatment, we considered that ^18^F-FDG-PET was superior to ^123^I-MIBG scintigraphy because ^18^F-FDG-PET images were not affected by the cell differentiation induced by 13-*cis*-RA treatment in our case. Thus, ^18^F-FDG-PET was useful for determining the treatment response and outcomes.

We have reported a case of residual neuroblastoma treated with differentiation-inducing 13-*cis*-RA therapy. Different results were produced with ^18^F-FDG-PET and ^123^I-MIBG scintigraphy. The cessation of 13-*cis*-RA treatment was based on ^18^F-FDG-PET findings and there has been no relapse for 3 years.

## INTRODUCTION

13-*cis*-retinoic acid (13-*cis*-RA) is a well-characterized differentiation agent used to treat neuroblastoma, and its continuous administration to high-risk neuroblastoma patients improves their prognoses.^1–4^ It is also used as a second-line treatment for residual and recurrent neuroblastoma.^5^ 13-*cis*-RA is usually administered for 14 days at a maintenance dose of 160 mg/m^2^, followed by 14 days of rest.^1–3^

However, the duration of 13-*cis*-RA treatment for residual or recurrent neuroblastoma remains controversial. In assessing the completion of 13-*cis*-RA treatment, it remains unclear whether to give priority to the findings of iodine-123 metaiodobenzylguanidine (^123^I-MIBG) scintigraphy or ^18^F-fluorodeoxyglucose positron emission tomography (^18^F-FDG-PET) when these are contradictory.

## CASE REPORT

**FIGURE 1:**
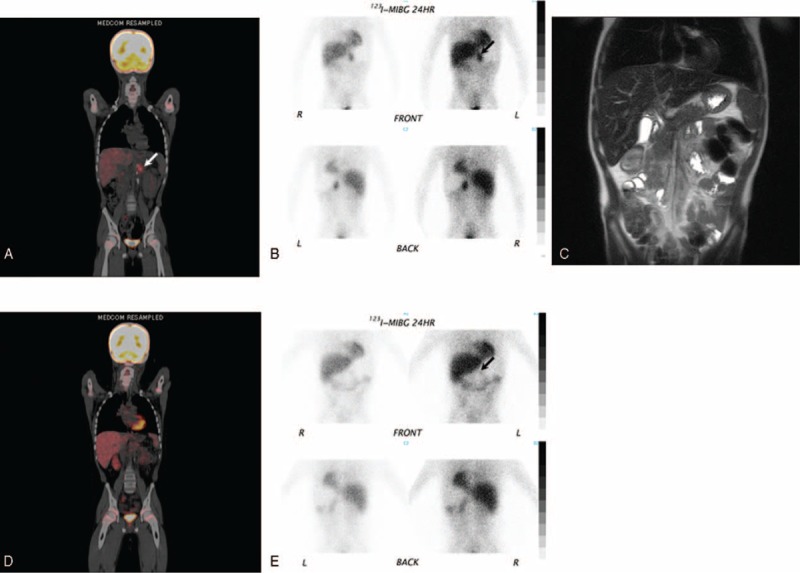
Images of an abdominal mass in an 8-year-old boy. (A) Coronal fused ^18^F-FDG-PET and CT before 13-*cis*-RA treatment. ^18^F-FDG accumulation is indicated by the arrow. (B) Planar ^123^I-MIBG scintigraphy before 13-*cis*-RA treatment. Tracer accumulation is indicated by the arrow. (C) No residual tumor is observed on T2-weighted coronal MRI acquired before 13-*cis*-RA treatment. (D) No ^18^F-FDG accumulation is observed 18 months after the initiation of 13-*cis*-RA treatment. (E) Faint ^123^I-MIBG uptake (arrow) is seen 18 months after the initiation of 13-*cis*-RA treatment. CT = computed tomography, 13-*cis*-RA = 13-*cis*-retinoic acid, ^18^F-FDG-PET = ^18^F-fluorodeoxyglucose positron emission tomography, ^123^I-MIBG = iodine-123 metaiodobenzylguanidine, MRI = magnetic resonance imaging.

An 8-year-old boy presented with a large abdominal mass. The patient had elevated levels of urinary vanillyl mandelic acid (VMA, 448.2 μg/mg Cr), homovanillic acid (HVA, 127.8 μg/mg Cr), and neuron-specific enolase (NSE, 590 pg/mL). The histology of the abdominal tumor revealed a neuroblastoma, which was poorly differentiated, had a high mitosis-karyorrhexis index, an unfavorable histology, and no *MYCN* gene amplification.^6,7^ The dissemination of metastases in the thoracic vertebrae prompted a diagnosis of stage 4 neuroblastoma. The patient was treated according to the protocol of the Japan Neuroblastoma Study Group for high-risk patients: 6 cycles of chemotherapy (cyclophosphamide, vincristine, pirarubicin, and cisplatin), followed by irradiation of the abdominal cavity, surgical resection of the adrenal gland, and megatherapy (melphalan, etoposide, and carboplatin) accompanied by autologous peripheral blood stem-cell transplantation. Following treatment, the patient's VMA, HMA, and NSE levels returned to the normal ranges, and no residual abdominal mass was detected with magnetic resonance imaging (MRI) (Figure 1C). However, the obvious accumulation of tracer on ^18^F-FDG-PET (Figure 1A, arrow) and ^123^I-MIBG scintigraphy (Figure 1B, arrow) indicated that viable neuroblastoma cells were still present. Analysis of a biopsy taken from around the area of ^123^I-MIBG accumulation during a second-look operation revealed that no residual tumor but live neuroblastoma cells were still present.

As treatment for the residual tumor cells, 13-*cis*-RA (160 mg/m^2^) was administered for 14 days, followed by 14 days of rest. Erythema, oral mucosal inflammation,^1^ and bone-marrow edema developed,^8^ although the erythema and inflammation receded during the 14-day rest period. 13-*cis*-RA treatment was discontinued 18 months after its initiation because ^18^F-FDG/PET showed no abnormal ^18^F-FDG uptake (Figure 1D), and the patient's VMA, HVA, and NSE levels were within the normal ranges, although ^123^I-MIBG revealed faint tracer accumulation (Figure 1E, arrow). Complete remission was still evident 3 years after the discontinuation of 13-*cis*-RA treatment.

## DISCUSSION

13-*cis*-RA is a differentiation agent for neuroblastoma cells. Randomized trials of 13-*cis*-RA treatment following myeloablative chemotherapy have shown that it reduces the incidence of relapse.^1–3^ However, the mechanism of action, treatment modalities, and the optimal duration of 13-*cis*-RA treatment for residual neuroblastoma following myeloablative chemotherapy remain controversial. Because 13-*cis*-RA is reported to have numerous adverse effects, treatment cannot proceed indefinitely and the optimal time for its discontinuation must be identified.^1,8–13^

In the present case, both ^123^I-MIBG and ^18^F-FDG detected active residual neuroblastoma cells before 13-*cis*-RA treatment (tracer accumultion is visible in Figure 1A, B), although no tumor was visualized with MRI (Figure 1C) or during a second-look operation. Eighteen months after the initiation of treatment, no ^18^F-FDG accumulation was visible (Figure 1D), suggesting the resolution of the active neuroblastoma cells. However, ^123^I-MIBG accumulation had not entirely disappeared at this time (Figure 1E), but we considered that the residual faint uptake of ^123^I-MIBG was caused by the catecholamine produced during the differentiation of the residual neuroblastoma cells into ganglioneuroma cells during 13-*cis*-RA treatment.^14,15^ On this basis, we decided that 13-*cis*-RA treatment could be safely terminated. No relapse has occurred in the 3 years since 13-*cis*-RA treatment.

Although ^123^I-MIBG scintigraphy-negative neuroblastoma has been reported,^16,17^^123^I-MIBG scintigraphy is a valuable tool for the diagnosis and staging of neuroblastoma, and is generally considered a sensitive and useful examination for this tumor.^14,15,18–20^ However, Brans et al^21^ argued that the degree of differentiation cannot be predicted by ^123^I-MIBG scintigraphy. Our case report confirms that ^18^F-FDG-PET is superior to ^123^I-MIBG scintigraphy for evaluating residual neuroblastoma activity following 13-*cis*-RA treatment, as ^18^F-FDG-PET images were not affected by the cell differentiation that occurs during this treatment. That our patient was still in remission 3 years after 13-*cis*-RA treatment shows that our diagnosis was correct.
